# Analysis of Corneal Spherical Aberrations in Chinese Bilateral Ectopia Lentis Patients

**DOI:** 10.3389/fmed.2021.736686

**Published:** 2021-11-19

**Authors:** Jiahui Chen, Yating Tang, Qinghe Jing, Yi Lu, Yongxiang Jiang

**Affiliations:** ^1^Department of Ophthalmology and Vision Science, Eye Ear Nose and Throat Hospital of Fudan University, Shanghai, China; ^2^National Health Commission (NHC) Key Laboratory of Myopia (Fudan University), Laboratory of Myopia, Chinese Academy of Medical Sciences, Shanghai, China; ^3^Key Laboratory of Visual Impairment and Restoration of Shanghai, Shanghai, China

**Keywords:** corneal spherical aberration, ectopia lentis, Marfan syndrome, corneal asphericity, aging

## Abstract

**Purpose:** To analyze the anterior, posterior, and total corneal spherical aberrations (ASA, PSA, and TSA) in patients with Chinese bilateral ectopia lentis (EL).

**Methods:** A cross-sectional study was conducted to evaluate corneal spherical aberration (CSA) using a Pentacam system at the 6-mm optical zone. Axial length, keratometry, astigmatism, and corneal asphericity were also determined.

**Results:** This study included 247 patients (420 eyes) with a mean age of 18.1 years. The values of ASA, PSA, and TSA were 0.136 ± 0.100 μm, −0.118 ± 0.030 μm, and 0.095 ± 0.095 μm, respectively. In the EL patients with Marfan syndrome (MFS), ASA and TSA were significantly lower than in the non-MFS patients (0.126 ± 0.094 μm vs. 0.155 ± 0.107 μm, *P* = 0.004 for ASA; 0.085 ± 0.091 μm vs. 0.114 ± 0.099 μm, *P* = 0.003 for TSA), whereas PSA was not significantly different (*P* = 0.061). The values of ASA and TSA were significantly higher in the patients with EL aged ≥ 40 years old than in younger patients, whereas ASA and PSA were lower in patients aged <10 years old than in older patients (all *P* < 0.05). In the multiple linear regression analysis, age, keratometry, astigmatism, anterior asphericity, higher-order aberration (HOA), and lower-order aberration (LOA) were positively or negatively correlated with TSA in the patients with EL (*r* = 0.681, *P* < 0.001).

**Conclusions:** Corneal spherical aberration was low in the patients with EL especially for MFS and tended to increase with aging. Preoperatively, individual measurement of CSA was necessary for bilateral EL patients with MFS.

## Introduction

Ectopia lentis (EL), which is dislocation or subluxation of the crystalline lens of the eye, is a condition that could occur metabolically or idiopathically. Metabolic syndromes include Marfan syndrome (MFS), homocystinuria, and Weill–Marchesani syndrome (1). EL caused by the weakness or loss of zonule can shift off the patient's visual axis and lead to a more spherical lens, inducing internal and spherical aberrations (SA) (2).

As a rotationally symmetric higher-order optical aberration, SA contributes to the deterioration of image quality and photic complaints (3–5). Prior studies have shown a marked variability in the extent of SA, not only among different ethnic groups with or without cataract, but also between eyes with different refractive states (6–10). CSA was reported to be ~+0.27 μm at a diameter of 6 mm, with a large SD of 0.10 μm (8). It was compensated for by the negative SA of the crystalline lens (11, 12) and had a statistically significant age-related change in general populations (13). Our previous study revealed that patients with MFS of EL with corneal optical properties had a significantly lower value of CSA than in healthy subjects as a control group (14). However, baseline wavefront data for CSA in the patients with bilateral EL between MFS and non-MFS remain unclear.

Clinical studies have reported that ignoring CSA in patients undergoing cataract or refractive lens surgery can significantly reduce their postoperative visual performance (3). Owing to recent advances in diagnostic and corrective methods, ophthalmologists have become more interested in CSA. Advances in surgical techniques and adjunct prosthetic devices made it possible for the in-the-bag placement and centration of intraocular lens (IOL) for patients with EL. With the removal of internal aberration of lens and the possibility of CSA compensation using an aspheric IOL, a better understanding of the characteristics of CSA will help ophthalmologists to improve the strategies for maintaining the quality of vision postoperatively in patients with EL. Therefore, we conducted a descriptive study to evaluate the characteristics of CSA (Zernike coefficient, Z 4 0) in patients with EL. We also investigated whether CSA correlated with age, axial length (AL), keratometry, corneal astigmatism, corneal asphericity, higher-order aberration (HOA), and lower-order aberration (LOA) determined using a Scheimpflug camera over a 6 mm diameter.

## Methods

We conducted a cross-sectional study of patients diagnosed with bilateral EL who were examined between October 2016 and October 2019 at the Eye and ENT Hospital of Fudan University, Shanghai, China. Slit-lamp examination revealed bilateral EL in 247 patients (494 eyes), including anterior or posterior dislocation of the lens and lens subluxation. The patients with EL were further divided into the non-MFS group (90 patients) and MFS group (157 patients), whose diagnosis was made according to the Ghent-2 criteria (15).

The study was conducted according to the tenets of the Declaration of Helsinki and was approved by the Human Research Ethics Committee of the Eye and ENT Hospital of Fudan University. Written informed consent was obtained from all patients (adults) and a parent and/or legal guardian (for minors) in this study.

All subjects were examined by two experienced doctors following the methods described before (13). All data of 420 eyes were collected with a Scheimpflug camera (Pentacam HR system, Oculus Inc., Wetzlar, Germany) and partial coherence interferometry (IOLMaster; Carl Zeiss Meditec, Jena, Germany). Only measurements marked “OK” in the quality specification for Pentacam were considered valid (16). Five valid readings of AL for IOLMaster were taken. A total of 74 eyes were excluded for the poor quality of Pentacam measures or a history of ocular surgery.

Measurements included AL determined using the IOLMaster, the mean keratometry (Km) of total corneal refractive power (TCRP), total corneal astigmatism (TCA), anterior spherical aberration (ASA), posterior spherical aberration (PSA), total spherical aberration (TSA), corneal asphericity (the *Q*-values), root mean square (RMS) of HOA, and RMS of LOA. The acquired CSA datasets were expanded with normalized Zernike polynomials and performed in the automatic mode with a 6-mm pupil scan diameter until accurate readings were obtained.

To analyze the differences in CSA with age, the patients with EL were divided into five age groups: G1 (95 patients; <10 years old), G2 (55 patients; 10–19 years old), G3 (45 patients; 20–29 years old), G4 (32 patients; 30–39 years old), and G5 (20 patients; ≥40 years old).

Categorical variables are presented as the absolute frequency (*n*) and relative frequency (%). The Kolmogorov–Smirnov test was used to assess the normality of the distribution of continuous variables. Where appropriate, continuous variables are presented as the mean ± SD. Student's *t*-test, the χ^2^ test, and Wilcoxon's rank-sum test (Mann–Whitney *U*-test) were used to compare data as appropriate. One-way ANOVA with *post-hoc* Bonferroni tests and the Kruskal–Wallis test were used to compare data among five age groups. Correlations between CSA and other parameters were assessed using Pearson's correlation test. Multiple linear regression analyses with the stepwise selection method were then performed to evaluate the associations between explanatory variables and TSA. In all analyses, *P-*values of < 0.05 were considered statistically significant. Statistical analyses were performed using SPSS software version 23.0 (IBM Corp., Armonk, NY, United States).

## Results

The study comprised 247 patients (420 eyes) of bilateral EL with a mean age of 18.1 ± 13.2 years (range: 3–56 years). The demographic and ocular biometric parameters in the patients with EL are presented in Table 1.

**Table 1:** Demographic and ocular characteristics in Chinese patients with bilateral ectopia lentis.

	**No. or mean ± SD**	**Range**
Subjects (eyes)	247 (420)	–
Sex (male/female)	129/118	–
Eyes (right/left)	205/215	–
Age (years)	18.1, 13.2	3 – 56
AL (mm)	25.72, 3.22	20.78 – 34.81
TCRP (D)	40.31, 1.67	36.4 – 46.1
TCA (D)	−1.59, 0.91	−4.1 to −0.1
ASA (μm)	0.136, 0.100	−0.177 – 0.435
PSA (μm)	−0.118, 0.030	−0.200 to −0.023
TSA (μm)	0.095, 0.095	−0.197 – 0.380
Asphericity F	−0.48, 0.19	−1.13 – 0
Asphericity B	−0.35, 0.19	−0.95 – 0.12
RMS (μm)	2.141, 0.820	0.405 – 4.472
RMS HOA (μm)	0.481, 0.183	0.168 – 1.166
RMS LOA (μm)	2.078, 0.820	0.334 – 4.398

*AL, axial length; TCRP, total corneal refractive power; TCA, total corneal astigmatism; ASA, anterior spherical aberration; PSA, posterior spherical aberration; TSA, total spherical aberration; F, front (i.e., anterior corneal surface); B, back (i.e., posterior corneal surface); RMS, root mean square; HOA, higher-order aberration; LOA, lower-order aberration; SD, standard deviation*.

The 277 eyes of the 157 patients with MFS and the 143 eyes of the 90 non-MFS patients had similar baseline parameters (Table 2). The mean ± SD age of the MFS and non-MFS groups was 17.9 ± 12.5 and 18.5 ± 14.3 years, respectively (*P* = 0.715). The mean values of ASA and TSA were significantly lower in the MFS group than in the non-MFS group measured at an optical zone of 6.0 mm (0.126 ± 0.094 vs. 0.155 ± 0.107 μm, *P* = 0.004 for ASA; 0.085 ± 0.091 vs. 0.114 ± 0.099 μm, *P* = 0.003 for TSA, Table 2), whereas PSA was not significantly different between the two groups (*P* = 0.061). The values of TSA were positive in 228 EL eyes with MFS (82.3%) and in 125 EL eyes without MFS (87.4%).

**Table 2:** The comparison of ocular characteristics in a cohort of Chinese patients with bilateral ectopia lentis divided into Marfan syndrome and non-Marfan syndrome.

**Groups**	**MFS**	**Non-MFS**	***P*-value**
Subjects (eyes)	157 (277)	90 (143)	
Sex (male/female)	83/74	46/44	0.790
Eyes (right/left)	134/143	71/72	0.804
Age (years)	17.9, 12.5	18.5, 14.3	0.715
AL (mm)	26.12, 3.39	24.95, 2.72	** <0.001**
TCRP (D)	40.00, 1.53	40.92, 1.77	** <0.001**
TCA (D)	−1.57, 0.89	−1.62, 0.95	0.616
ASA (μm)	0.126, 0.094	0.155, 0.107	**0.004**
PSA (μm)	−0.116, 0.029	−0.122, 0.032	0.061
TSA (μm)	0.085, 0.091	0.114, 0.099	**0.003**
Asphericity F	−0.50, 0.20	−0.45, 0.18	**0.003**
Asphericity B	−0.36, 0.20	−0.32, 0.17	**0.022**
RMS (μm)	2.133, 0.787	2.157, 0.882	0.779
RMS HOA (μm)	0.485, 0.183	0.474, 0.185	0.532
RMS LOA (μm)	2.069, 0.787	2.096, 0.881	0.746

*MFS, Marfan syndrome; AL, axial length; TCRP, total corneal refractive power; TCA, total corneal astigmatism; ASA, anterior spherical aberration; PSA, posterior spherical aberration; TSA, total spherical aberration; F, front (i.e., anterior corneal surface); B, back (i.e., posterior corneal surface); RMS, root mean square; HOA, higher-order aberration; LOA, lower-order aberration. Bold value: significant*.

**Figure 1:**
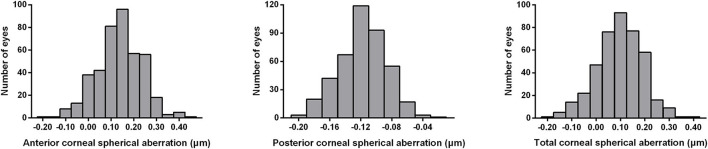
Distribution of anterior, posterior, and total spherical aberrations of the cornea measured by the Pentacam system in 420 eyes of patients with bilateral ectopia lentis. ASA, anterior spherical aberration; PSA, posterior spherical aberration; TSA, total spherical aberration.

A histogram of the distribution of CSA in the patients with EL is shown in Figure 1. It revealed an almost symmetrical distribution around the mean TSA value of 0.095 μm (range: −0.197–0.380 μm); 73.1% of these values were between 0.0 and 0.2 μm, 15.7% were < 0.0 μm, and 11.2% were > 0.2 μm.

In the patients with EL, the mean AL was 25.72 ± 3.22 mm (range: 20.78– 34.81 mm); the Km reading of total corneal refractive power was 40.31 ± 1.67 D (range: 36.4–46.1 D); and the mean total corneal astigmatism was −1.59 ± 0.91 D (range: −4.1 to −0.1 D, Table 1). AL, total corneal refractive power, and total corneal astigmatism were statistically different between the MFS and non-MFS groups (Table 2). Similarly, corneal asphericity (the *Q*-values) of the anterior and posterior surfaces was significantly different between the MFS and non-MFS groups (−0.50 ± 0.20 vs. −0.45 ± 0.18, *P* = 0.003 for the anterior surface; −0.36 ± 0.20 vs. −0.32 ± 0.17, *P* = 0.022 for the posterior surface, Table 2). However, no statistical difference was found for RMS, RMS HOA, and RMS LOA between the MFS and non-MFS groups (Table 2).

**Figure 2:**
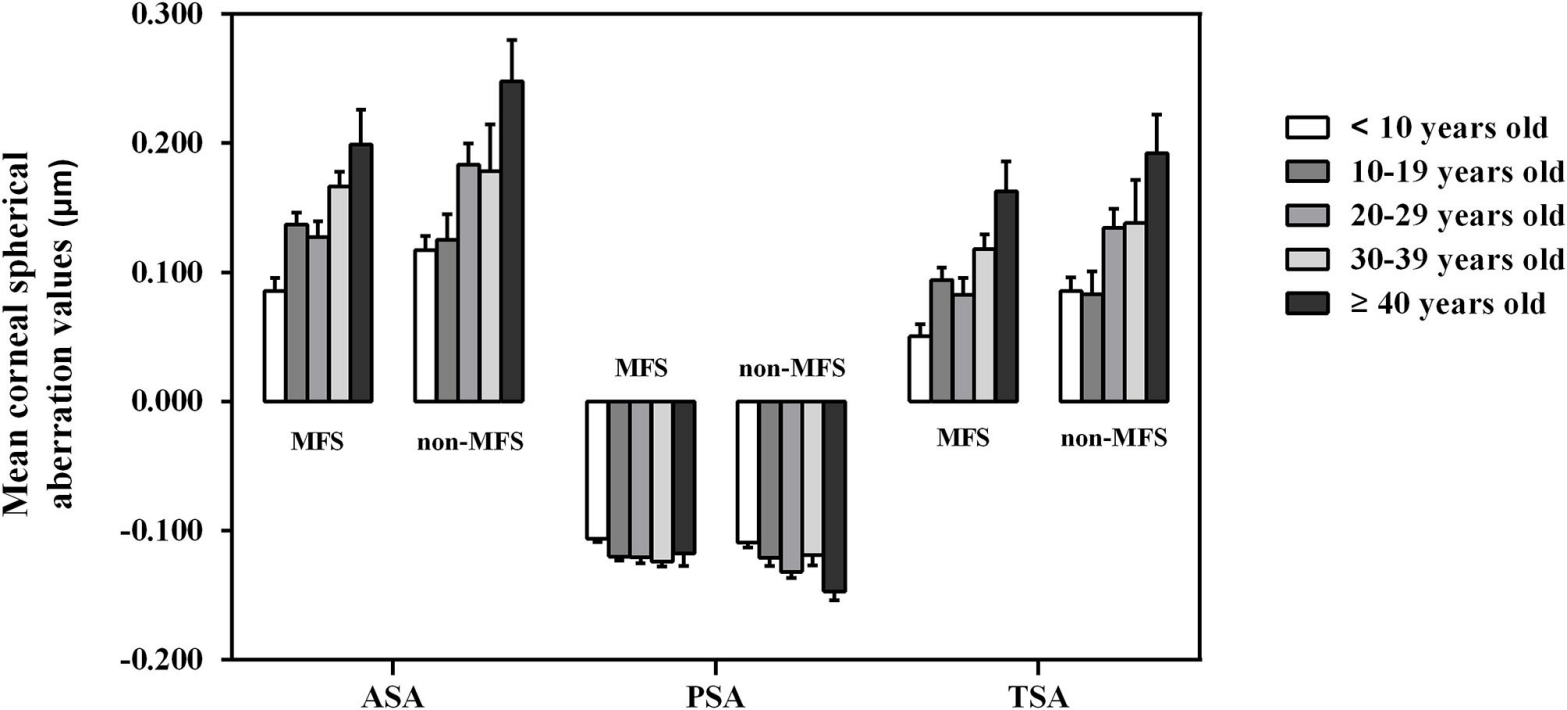
Comparisons of anterior, posterior, and total spherical aberrations of cornea among the five age groups of the patients with bilateral ectopia lentis divided by Marfan and non-Marfan syndrome groups. Error bars represent the standard error of the mean. ASA, anterior spherical aberration; MFS, Marfan syndrome; PSA, posterior spherical aberration; TSA, total spherical aberration.

To analyze potential factors that influence CSA, the values of ASA, PSA, and TSA were compared in the patients with EL divided by age, sex, and right or left eye. Of all 420 EL eyes tested, ASA and TSA differed significantly among the five age groups and were higher in G5 (20 patients, 34 eyes) than in the other four groups [G1 (95 patients, 153 eyes); G2 (55 patients, 97 eyes); G3 (45 patients, 79 eyes); and G4 (32 patients, 57 eyes); all *P* < 0.05, Table 3], whereas ASA and PSA were lower in G1 than in the four older groups (G2, G3, G4,and G5; all *P* < 0.05, Table 3). The values of CSA did not differ significantly for any part of the cornea between men and women or between the right and left eyes (all *P* > 0.05, Table 3). Comparisons of ASA, PSA, and TSA of cornea among the five age groups of the patients with EL divided by MFS and non-MFS groups are shown in Figure 2.

**Table 3:** Anterior, posterior, and total spherical aberration of the cornea with a 6-mm pupil size in patients with bilateral ectopia lentis divided by age, sex, and eye.

**Groups**	**N (patients/eyes)**	**ASA (μm)**	**PSA (μm)**	**TSA (μm)**
**Age (years)**
<10	95/153	0.098 ± 0.092^*^	−0.107 ± 0.026^*^	0.064 ± 0.088
10–19	55/97	0.134 ± 0.085	−0.120 ± 0.027	0.091 ± 0.084
20–29	45/79	0.148 ± 0.090	−0.125 ± 0.033	0.102 ± 0.090
30–39	32/57	0.169 ± 0.088	−0.123 ± 0.027	0.122 ± 0.086
≥40	20/34	0.226 ± 0.126^*^	−0.134 ± 0.036	0.179 ± 0.114^*^
*P*-value		** <0.001**	** <0.001**	** <0.001**
**Sex**
Male	129/214	0.132 ± 0.096	−0.117 ± 0.031	0.091 ± 0.093
Female	118/206	0.139 ± 0.103	−0.119 ± 0.029	0.098 ± 0.097
*P*-value		0.500	0.644	0.432
**Eye**
Right	–/205	0.133 ± 0.100	−0.118 ± 0.030	0.092 ± 0.095
Left	–/215	0.138 ± 0.099	−0.117 ± 0.030	0.097 ± 0.095
*P*-value		0.660	0.694	0.576

*ASA, anterior spherical aberration; PSA, posterior spherical aberration; TSA, total spherical aberration*.

* *P < 0.05, compared with the other four age groups. Bold value: significant*.

**Figure 3:**
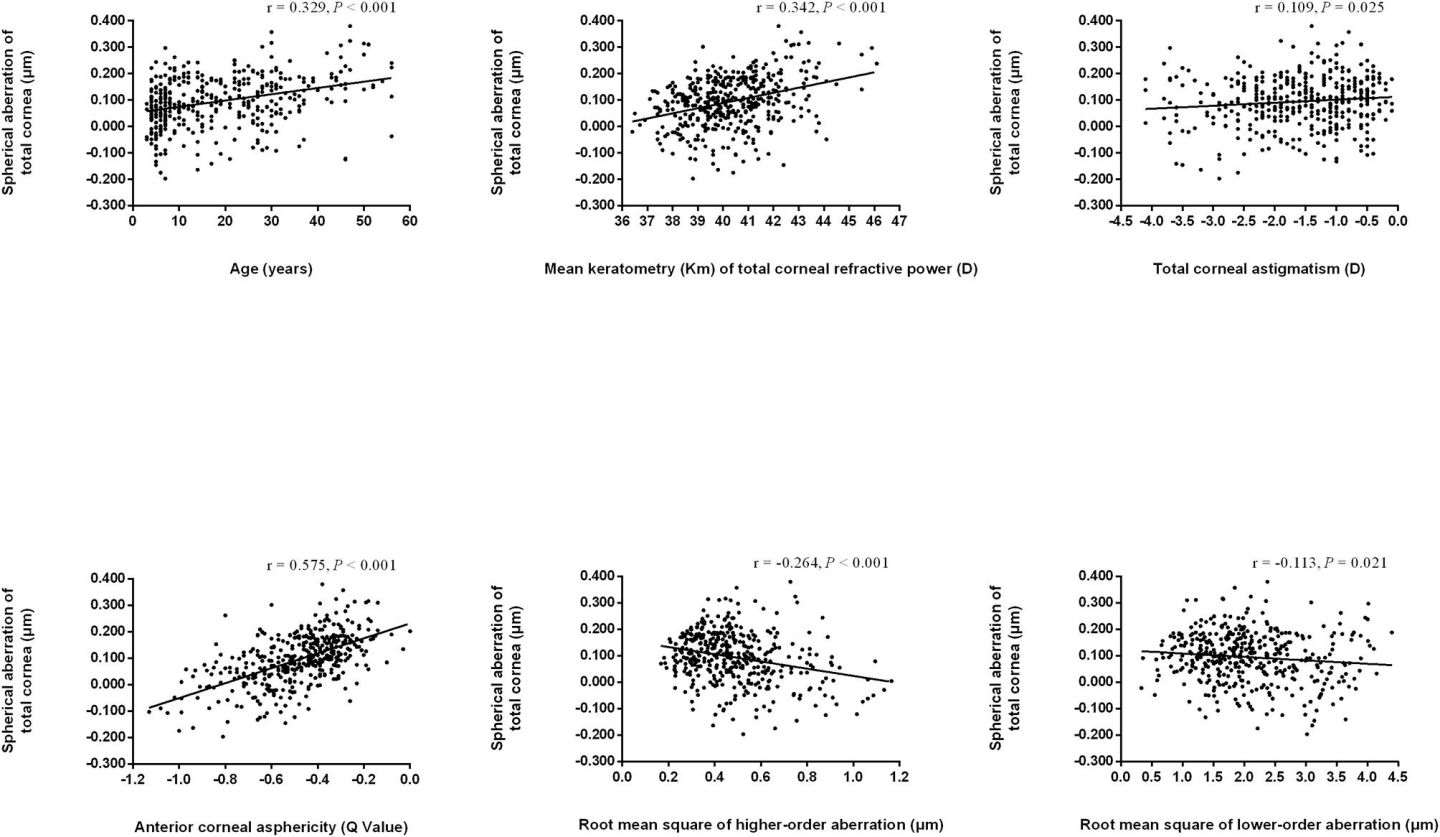
Scatter plots indicating correlations of total spherical aberration of the cornea with other biometric parameters. Regression lines are drawn when the regression analyses are statistically significant.

We found that TSA was positively correlated with age (*r* = 0.329, *P* < 0.001), total corneal refractive power (*r* = 0.342, *P* < 0.001), total corneal astigmatism (*r* = 0.109, *P* = 0.025), and corneal asphericity (*r* = 0.575, *P* < 0.001 for the anterior surface; *r* = 0.269, *P* < 0.001 for the posterior surface). However, TSA was negatively correlated with RMS (*r* = −0.125, *P* = 0.011), RMS HOA (*r* = −0.264, *P* < 0.001), and RMS LOA (*r* = −0.113, *P* = 0.021, Figure 3). Similarly, ASA was positively correlated with age, total corneal refractive power, total corneal astigmatism, and corneal asphericity and was negatively correlated with RMS, RMS HOA, and RMS LOA, whereas PSA was negatively correlated with age, total corneal refractive power, and corneal asphericity and was positively correlated with RMS, RMS HOA, and RMS LOA (data not shown).

The associations between TSA and the variables, including age, AL, total corneal refractive power, total corneal astigmatism, corneal asphericity, RMS, RMS HOA, and RMS LOA, were investigated using the multiple linear regression analysis. It revealed that the parameters, including age, total corneal refractive power, total corneal astigmatism, anterior asphericity, RMS HOA, and RMS LOA, were significantly associated with TSA in the patients with EL (*r* = 0.681, all *P* < 0.05; Table 4). However, no association was found between TSA and AL, RMS, or posterior corneal asphericity after adjusting for other factors in the patients with EL.

**Table 4:** Multiple linear regression analysis of factors associated with total corneal spherical aberration in patients with bilateral ectopia lentis.

	**Unstandardized coefficients (B)**	**Standardized coefficients (Beta)**	**95% CI for B**	***P*-value**
Age	0.001	0.169	0.001 – 0.002	** <0.001**
TCRP	0.010	0.184	0.006 – 0.015	** <0.001**
TCA	0.045	0.430	0.027 – 0.063	** <0.001**
Asphericity F	0.221	0.451	0.184 – 0.259	** <0.001**
RMS HOA	−0.196	−0.378	−0.250 to −0.141	** <0.001**
RMS LOA	0.063	0.540	0.039 – 0.086	** <0.001**

*TCRP, total corneal refractive power; TCA, total corneal astigmatism; F, front (i.e., anterior corneal surface); RMS, root mean square; HOA, higher-order aberration; LOA, lower-order aberration; CI, confidence interval. Bold value: significant*.

## Discussion

The quality of vision may deteriorate because of corneal aberrations. In recent years, ophthalmologists have gained a greater understanding of the impact of aberrations on vision owing to the increasing availability of wavefront-sensing devices in ophthalmic clinics (17, 18). Advances in surgical techniques and adjunct prosthetic devices mean in-the-bag placement and centration of IOL are usually successful in patients with EL (19). Based on the significant improvements in aspherical IOLs to eliminate CSA, a better understanding of the characteristics of CSA in patients with EL can improve the accuracy of IOL design and facilitate appropriate IOL selection. Therefore, we used the Pentacam HR system to assess CSA in patients with EL because very few studies have provided detailed descriptions of CSA.

The tilt decentration of the lens-induced HOAs can lead to deterioration in the quality of vision. Significantly, EL and the resultant spherophakia were very likely to contribute to the internal aberrations. After the subluxated lens was removed, CSA might contribute considerably to the deterioration of image quality. The positive SA would provide some protection against myopia progression (20). Our study revealed that the implantation of a neutral or low-negative aberration aspheric IOL would be preferable in patients with EL.

Visual quality following cataract surgery may show age-related deterioration in corneal aberrations. Although CSA was unaffected by age in some studies (11, 21, 22). later studies revealed a correlation with age when using specialized aberrometer in patients with cataract (6, 23, 24). Here, we found a positive correlation between age and TSA in patients with EL that might be attributed to an increased positive ASA. The steepening of the anterior corneal surface was correlated with age-related increases in ASA in some studies (5, 8, 25–27). Using Scheimpflug photography, Sicam et al. (28) observed an increase of CSA with advancing age because PSA was negative at a young age and became positive at an older age. However, we found PSA was negatively correlated with age. To make an accurate description of CSA in the patients with EL, the posterior corneal surface should not be ignored.

Age-related changes of CSA may affect the refractive postoperative outcomes of young children or older patients with EL. Kemraz et al. (13) found that CSA became more positive after 39 years of age. Similarly, in our patients with EL, TSA was higher in G5 (≥40 years old) than in the other four groups. What's more, ASA was lower in G1 (<10 years old) than in the four older groups. This increase of CSA in the older population may be attributed to the change in corneal asphericity from a prolate ellipsoid into a more spherical shape (13). In younger patients, the internal optics compensate for part of the corneal aberrations, leading to a lower SA. Clinical evaluation is required for these patients to improve the quality of vision following cataract surgery.

The mean values of ASA and TSA were significantly lower in the MFS group than in the non-MFS group measured at an optical zone of 6.0 mm. Although not statistically significant, the value of PSA was less negative in MFS eyes. In our previous study, patients with MFS with decreased keratometry were found to have lower CSA than health subjects (13). The steeper central region and flatter periphery may reduce the number of SAs in the eye. In the management of patients with bilateral EL, the CSA of MFS is relatively small, which has significance for selecting IOL.

Few studies have reported the correlation between CSA and ocular biometric parameters in patients with EL. In some studies, CSA significantly correlated with AL but not corneal curvature in the general population (6, 9); however, Beiko et al. found a very weak correlation between CSA and corneal central keratometry parameters (5). In the current study, TSA tended to increase with increases in keratometry. Longitudinal studies have shown that the corneal curvature of myopic children may flatten between the ages of 9 and 12 years (29), indicating that CSA may decrease in this period. Multiple linear regression analysis revealed that age and total corneal refractive power were positively associated with TSA in the patients with EL. Therefore, the selection of aspherical IOL in young patients with EL deserved to be deliberated.

In our study, corneal asphericity (the *Q*-value) was directly proportional to TSA with a correlation coefficient of 0.575 for the anterior corneal surface. As anterior corneal asphericity became more positive, TSA increased. The corneal asphericity described the rate of curvature variation of the cornea from its center to the periphery and separated more pronounced corneal flattening. It is possible that eyeball elongation and corneal thinning have particular consequences on the junction between the peripheral cornea and the sclera, affecting the values of CSA synergistically or oppositely (30). The determination of corneal asphericity, which characterizes the variation between the central and the peripheral curvature of the cornea, would provide a detailed information for CSA in the patients with EL.

To our knowledge, there were few studies to evaluate the characteristics of CSA, as well as the correlations between CSA and ocular biometric parameters in patients with EL. One limitation was the different visual and refractive status of participants. The poor preoperative fixation stability in the patients with EL with a long AL means that the Scheimpflug analysis may exhibit limited acuity for corneal biometrics. Finally, the analysis of internal aberrations would be much more appropriate to add information regarding the TSA of the eye in patients with EL; further study should be conducted in the future.

In conclusion, the value of TSA was low in this cohort of patients with EL, especially for patients with MFS. Implanting a neutral or low-negative SA aspheric IOL was recommended for patients with EL. Taking into consideration the age-related change and correlation with ocular biometric parameters, we believe preoperatively individual measurement of CSA is necessary for patients with bilateral EL, and the results should guide the selection of aspheric IOL for implantation in the patients with EL.

## Data Availability Statement

The original contributions presented in the study are included in the article/supplementary material, further inquiries can be directed to the corresponding author/s.

## Author Contributions

JC and YJ were involved in conception and design. JC was responsible for the analysis and interpretation of results and wrote the first draft of the manuscript. YT and QJ participated in the data collection. YL and YJ revised the manuscript and supervised the study. All authors reviewed and approved the final manuscript.

## Funding

This work was supported by the National Natural Science Foundation of China (Grant No. 81770908), the National Key R&D Program of China (2018YFC0116000), and the Shanghai Science and Technology Commission (18411965200).

## Conflict of Interest

The authors declare that the research was conducted in the absence of any commercial or financial relationships that could be construed as a potential conflict of interest.

## Publisher's Note

All claims expressed in this article are solely those of the authors and do not necessarily represent those of their affiliated organizations, or those of the publisher, the editors and the reviewers. Any product that may be evaluated in this article, or claim that may be made by its manufacturer, is not guaranteed or endorsed by the publisher.
